# Rupture of Ovarian Pregnancy in a Woman with Low Beta-hCG Levels

**DOI:** 10.1155/2012/213169

**Published:** 2012-11-11

**Authors:** Serena Resta, Eliana Fuggetta, Federica D'Itri, Simona Evangelista, Adele Ticino, Maria Grazia Porpora

**Affiliations:** Department of Obstetrics, Gynaecology and Urology, Sapienza University of Rome, Viale del Policlinico 155, 00161 Rome, Italy

## Abstract

*Background*. Ovarian pregnancy is a rare form of ectopic pregnancy. It is often difficult to distinguish from tubal pregnancy, and diagnosis and management are frequently a challenge. *Case*. A 33-year-old nulligravida woman presented with light vaginal bleeding and bilateral lower quadrants abdominal pain. Beta-human chorionic gonadotropin (beta-hCG) level (592 mUI/mL) and clinical and ultrasound (US) findings were suspicious for tubal pregnancy. On the third day, despite beta-hCG decrease (364 mUI/mL), she complained of severe pain in the lower abdomen, and physical examination revealed abdominal rebound tenderness. US showed a large amount of fluid in the abdominal cavity. Because of the unstable clinical condition, emergency laparoscopy and resection of left ovarian ectopic pregnancy were performed. Histology confirmed ovarian gestation. *Conclusion*. This case shows that ectopic pregnancy rupture may occur despite low levels of beta-hCG. Hemoperitoneum is not contraindication to laparoscopy.

## 1. Introduction

Ovarian pregnancy is one of the rarest variants of ectopic implantation, and incidence is estimated to be 3% of diagnosed ectopic pregnancies [1]. The diagnosis of ovarian pregnancy is difficult. We report a case of ruptured ovarian pregnancy misdiagnosed as tubal pregnancy, which caused significant hemoperitoneum and was successfully treated by conservative laparoscopy.

## 2. Case Presentation

**Figure 1:**
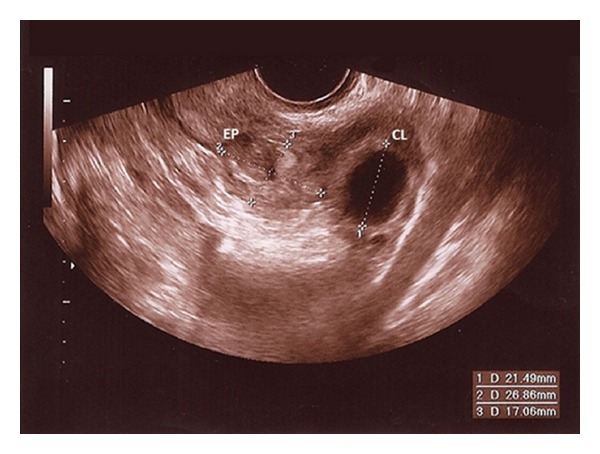
Transvaginal gray-scale US image of left adnexa reveals a 25 mm hyperechoic mass with a central hypoechoic shadow, suggestive of a tubal pregnancy. ES: ectopic sac; CL: corpus luteum.

A 33-year-old nulliparous woman presented to the gynecologic emergency room of our Department complaining of light vaginal bleeding and bilateral lower quadrants abdominal pain without signs and symptoms of circulatory instability. She reported a history of 5 weeks of amenorrhea, and her previous menstrual cycles were regular. She had no history of sexually transmitted disease or pelvic inflammatory disease, nor previous gynecological surgery. Physical examination revealed initial blood pressure 110/70 mmHg, pulse 80 beats/min, and body temperature 37.5°C. On speculum examination there was a small amount of blood in the vagina but the cervical os was closed. On bimanual clinical examination she was found to have a normal size of anteverted uterus and no cervical motion tenderness. The left adnexa was tender to palpation but no adnexal mass was appreciated. Urine pregnancy test was positive, and the serum beta-chorionic gonadotropin (beta-hCG) level was 592 mUI/mL. Transvaginal ultrasound (US) examination showed an empty uterus, corpus luteum in the left ovary and a 25 mm hyperechoic mass with a central hypoechoic shadow in the left adnexa, suggestive of a tubal pregnancy (Figure 1). A structure simulating yolk sac or fetal pole was not found. The right adnexa was normal, and there was no sign of free fluid in the abdominal cavity. Her initial laboratory results were: White Blood Cells (WBC): 4.6 × 10^9^/L, Hemoglobin (Hb): 12 g/dL, Hematocrit (Hct): 34%, Platelets (Plt): 216 × 10^9^/L, and Red Blood Cells (RBC): 4.2 × 10^12^/L. The patient was admitted to the Gynecological ward with a diagnosis of suspected tubal pregnancy. Daily routine blood examinations, including serum beta-hCG, and US were performed showing that the patient was in a stable clinical condition.

**Figure 2:**
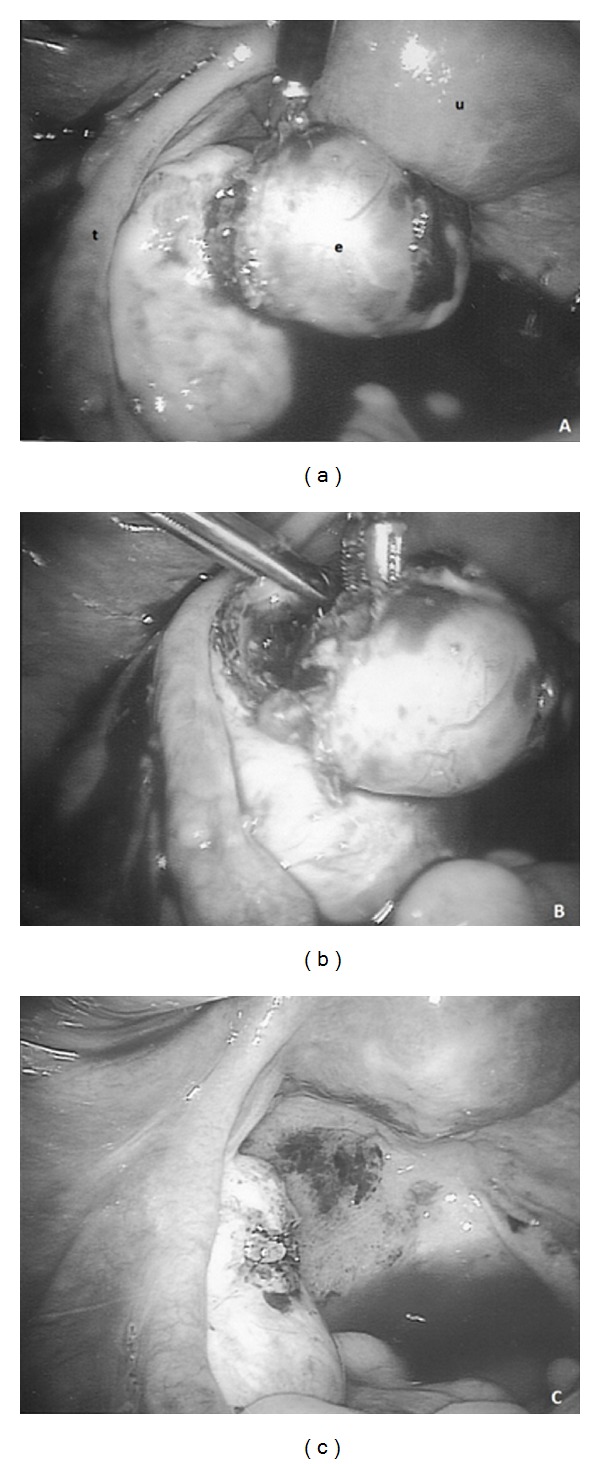
The laparoscopic treatment of the ovarian pregnancy: (a) at the beginning of the procedure: normal aspect of the uterus (u) and left fallopian tube (t), ruptured ectopic pregnancy in the left ovary (e); (b) gestational sac shelled out of the encasing ovarian tissue; (c) appearance of the left ovary after removal of ectopic pregnancy.

On the third day of hospitalization, despite a beta-hCG decrease to 364 mIU/mL, the patient complained of severe pain in the lower abdomen, and there was abdominal rebound tenderness during physical examination. The patient's pulse rate was 90/min, and blood pressure 95/60 mmHg. Hb levels dropped to 8.5 g/dL, the number of erythrocytes reduced to 3.17 × 10^12^/L, and Hct to 23.5%. Transvaginal US showed a large amount of fluid in the cul-de-sac and abdominal cavity. The patient was taken to the operating room with a presumptive diagnosis of a ruptured ectopic pregnancy and hemoperitoneum. Emergency laparoscopy was performed. During laparoscopy, 600 mL of fresh blood along with dark blood clots were evacuated from the abdominal cavity. The left fallopian tube appeared to be normal. Active bleeding was observed from the left ovary, where a hemorrhagic mass suggestive of ruptured ectopic pregnancy (5 cm × 4.5 cm × 3.5 cm) was present. The gestational sac was easily shelled out of the encasing ovarian tissue (Figure 2). Pathologic analysis confirmed a left ovarian gestation with presence of necrotic chorionic villi and a corpus luteum within the ovarian tissue. After laparoscopy, hemoglobin value was 8.1 g/dL, and no blood transfusion was needed. The patient had an uneventful postoperative course and was discharged two days later.

## 3. Discussion

Ovarian pregnancy is a rare variant of ectopic pregnancy. Early diagnosis of ovarian pregnancy is necessary in order to avoid more serious complications and emergency invasive procedures [2]. However, preoperative diagnosis remains challenging, and it is diagnosed generally during surgery [3]. Correct diagnosis of ectopic pregnancy can often be made on the basis of patient history, signs and symptoms, serum beta-hCG levels, and pelvic US examination. In our case the ovarian pregnancy was misdiagnosed as a tubal pregnancy, as US was not useful for distinguishing between ovarian and tubal pregnancy. Low beta-hCG levels may be misleading, and tubal ruptures have been reported in cases with decreasing beta-hCG [4]. An accurate differential diagnosis is important in ectopic pregnancies as patient management often differs depending on the type and the exact location of the pregnancy [5]. Medical therapy with methotrexate was not a possible option due to the occurrence of massive bleeding. Generally, in case of hemoperitoneum most surgeons prefer to perform laparotomy. Few cases of laparoscopic treatment in women with hemoperitoneum have been reported [6]. In our case the ovarian pregnancy was easily removed laparoscopically after evacuation of blood from the abdominal cavity. This conservative technique allowed preservation of the ovary and reproductive capability.

In conclusion, ovarian pregnancy is a rare event that is difficult to detect, and a quantitative beta-hCG level may be an unreliable predictor of ectopic pregnancy evolution. Hemoperitoneum is not always a contraindication to laparoscopy.
